# ﻿Ex situ population of the Harpy Eagle and its potential for integrated conservation

**DOI:** 10.3897/zookeys.1083.69047

**Published:** 2022-01-25

**Authors:** Marcos José de Oliveira, Francisca Helena Aguiar-Silva, Wanderlei de Moraes, Tânia Margarete Sanaiotti, Aureo Banhos, Nei Moreira

**Affiliations:** 1 Itaipu Binacional, Av. Tancredo Neves, 6731, Itaipu “A”, 85856–970, Foz do Iguaçu, Paraná, Brazil Itaipu Binacional Foz do Iguaçu Brazil; 2 Universidade de São Paulo – USP, Centro de Energia Nuclear na Agricultura – CENA, Av. Centenário, 303, São Dimas, 13400–970, Piracicaba, Brazil Projeto Harpia Manaus Brazil; 3 Projeto Harpia (Harpy Eagle Project), Av. André Araújo, 2936, Aleixo, 69067–375, Manaus, Amazonas, Brazil Universidade de São Paulo Piracicaba Brazil; 4 Médico veterinário autônomo – Rua do Angico, 186, Itaipu B, 85867–100, Foz do Iguaçu, Paraná, Brazil Médico veterinário autônomo – Rua do Angico Foz do Iguaçu Brazil; 5 Instituto Nacional de Pesquisas da Amazônia – INPA, Coordenação de Biodiversidade, Av. André Araújo, 2936, Aleixo, 69067–375, Manaus, Amazonas, Brazil Instituto Nacional de Pesquisas da Amazônia Manaus Brazil; 6 Universidade Federal do Espírito Santo – UFES, Departamento de Biologia, Centro de Ciências Exatas, Naturais e da Saúde, Alto Universitário, s/n°, Guararema, 29500–000, Alegre, Espírito Santo, Brazil Universidade Federal do Espírito Santo Alegre Brazil; 7 Universidade Federal do Paraná – UFPR, Programa de Pós-Graduação em Zoologia, Departamento de Biociências, Rua Pioneiro, 2153, Jardim Dallas, 85950–000, Palotina, Paraná, Brazil Universidade Federal do Paraná Palotina Brazil

**Keywords:** Birds of prey, captive breeding, *Harpiaharpyja*, threatened species

## Abstract

A main priority in conservation is the protection of species in their natural habitat. However, ex situ management of threatened species is a recognised strategy of conservation. Harpy Eagles (*Harpiaharpyja*) are removed from the wild due to illegal capture, nest tree destruction, or other conflict sources. This study presents a review of the current ex situ Harpy Eagle populations in Brazil and worldwide, including information on the origin, sex, and year of entrance or year of birth under human care. Worldwide, until 2020 there were 205 Harpy Eagles in 77 different facilities in 16 countries, with 40 institutions in Brazil and 37 in other countries. The largest ex situ Harpy Eagle population is maintained in Brazil, with 139 individuals (75 females and 64 males) in 40 institutions. Of these institutions, there were 24 zoos, seven conservation breeding centres, six commercial breeders, two wildlife shelters, and one wildlife sorting centre. In Brazil, 62% (*n* = 86) of the individuals were hatched in the wild and 38% (*n* = 53) were bred in captivity under human care; for the wild individuals, only 73% (*n* = 64) have a known state of origin, with the majority from Pará state. This investigation provided relevant information to establish an ex situ demographic database. These individuals may potentially constitute a genetically and demographically viable safety population for future conservation strategies, as well as a source for research and education applied to Harpy Eagle integrated conservation.

## ﻿Introduction

Conservation actions for endangered bird populations involve the maintenance of the natural habitat, with protection of the nests and offspring until they are mature enough to disperse (Butchart et al. 2006; Pacifico et al. 2014). The Harpy Eagle (*Harpiaharpyja* Linnaeus, 1758) is a bird of prey and a top predator with a large carrying capacity (Voous 1969). With a low population density and slow reproductive rate in nature, the Harpy Eagle relies on conservation action plans and is the subject of extensive research projects (Soares et al. 2008; Brasil 2014b; Aguiar-Silva et al. 2015; Sanaiotti et al. 2015; Watson et al. 2016; Oliveira 2018).

The Harpy Eagle is globally classified as a Vulnerable species (Bird Life International 2021) and is listed in Appendix I of CITES (2017). In Brazil, which has the largest population, it has been classified as Vulnerable to extinction since 2014 due to the loss of habitat and removal of individuals from nature (Brasil 2014a; Banhos et al. 2018). However, in the evaluation of the Brazilian Atlantic Forest states, the population status of the Harpy Eagle is more concerning categories, being considered Endangered in Rio de Janeiro state (Alves et al. 2000), Critically Endangered in São Paulo, Paraná (Silveira et al. 2009) and Espírito Santo states (Duca et al. 2019), and probably Extinct in Rio Grande do Sul state (Bencke et al. 2003). However, in the far South of Brazil, there were recently documented records of an adult and a juvenile eagle in the region of Turvo State Park (Meller and Guadagnin 2016; Kuhn 2018). The first Harpy Eagles in the care of a zoo in Brazil were reported during the 1890’s (Sanjad et al. 2012; Pais 2013), but in the last few decades, they have been frequently removed from nature by anthropogenic actions (Trinca et al. 2008; DeLuca 2012; Silva et al. 2013; Freitas et al. 2014; Gusmão et al. 2016; Gusmão et al. 2020), and many have been destined for zoos (This study, Table 1).

**Table 1.** Harpy Eagle (*Harpiaharpyja*) ex situ population in Brazil in 2020. Regions: N–North, NE–Northeast, MW–Midwest, SE–Southeast, and S–South. Management category: Zoo, ConsBr–Conservationist Breeder, ComBr–Commercial Breeder, WSC–Wildlife Sorting Centre, WS–Wildlife Shelter. IBAMA–Brazilian Institute of Environment and Renewable Natural Resources (Instituto Brasileiro do Meio Ambiente e dos Recursos Naturais Renováveis). For state acronyms, see Materials and methods.

#	Institution keeper	Region	State	City	Administration Type	Management Category	Origin of Birth				Breeding Period	Replied Survey
							Wild		Bred in Captivity			
							♂	♀	♂	♀		
1	Parque Zoobotânico Getúlio Vargas	NE	BA	Salvador	State	Zoo	0	1	0	1	2017	Yes
2	Criadouro Comercial Haras Claro	NE	CE	Caucaia	Private	ComBr	1	1	0	0	–	Yes
3	Criadouro Comercial Sítio Tibagi	NE	CE	Guaramiranga	Private	ComBr	1	1	0	0	2006–2018	Yes
4	Criadouro Conservacionista Ararajuba do Ipê – Gilrassic Park	NE	MA	Santa Inês	Private	ConsBr	0	3	0	0	–	–
5	Parque Estadual Dois Irmãos	NE	PE	Recife	State	Zoo	0	1	0	0	–	Yes
6	Parque Estadual Zoobotânico de Piauí	NE	PI	Teresina	State	Zoo	0	1	0	0	–	Yes
7	Parque dos Falcões	NE	SE	Itabaiana	Private	ConsBr	1	1	0	0	–	No
8	Centro de Triagem de Fauna Silvestre- IBAMA	N	AC	Rio Branco	Federal	WSC	0	2	0	0	–	–
9	Parque Urbano Estadual Chico Mendes	N	AC	Rio Branco	State	Zoo	0	1	0	0	–	Yes
10	Zoológico do Centro de Instrução de Guerra na Selva	N	AM	Manaus	Federal	Zoo	0	2	0	0	–	Yes
11	RPPN Revecom	N	AP	Santana	Private	WS	0	1	0	0	–	Yes
12	Criadouro Fazenda Paricuiã	N	PA	Terra Alta	Private	ConsBr	1	0	0	0	–	–
13	Fundação Zoobotânica de Marabá	N	PA	Marabá	Private	Zoo	1	1	0	0	–	No
14	Mantenedouro de Fauna Silvestre Santo Antonio	N	PA	Ananindeua	Private	WS	0	1	0	0	–	–
15	Parque Zoobotânico Emilio Goeldi	N	PA	Belém	Federal	Zoo	0	1	0	0	–	Yes
16	Parque Zoobotânico Vale	N	PA	Carajás	Private	Zoo	1	0	0	0	2015	Yes
17	Zoológico Faculdade da Amazônia – UNAMA	N	PA	Santarém	Private	Zoo	1	1	0	0	–	No
18	Criadouro Conservacionista Spazen	MW	DF	Brasília	Private	ConsBr	1	0	1	0	–	Yes
19	Fundação Jardim Zoológico de Brasília	MW	DF	Brasília	Foundation	Zoo	1	1	0	0	–	Yes
20	Criadouro Científico Instituto Onça Pintada	MW	GO	Mineiros	Private	ConsBR	0	0	1	2	–	Yes
21	Zoológico de Goiânia	MW	GO	Goiânia	County	Zoo	0	1	0	0	–	No
22	Zoopark da Montanha	SE	ES	Marechal Floriano	Private	Zoo	0	1	0	1	–	No
23	CRAX Sociedade de Pesquisa de Fauna Silvestre	SE	MG	Contagem	Private	ConsBr	3	6	9	3	1999–2005	No
24	Centro de Reprodução de Falconiformes e Falcoaria Ltda- Criadouro Cerefalco	SE	MG	Patrocínio	Private	ComBr	1	0	1	2	–	Yes
25	Criadouro Científico de Aves de Rapina Pró-Raptors	SE	MG	Brumadinho	Private	ConsBr	4	3	0	0	–	Yes
26	Criadouro Global Falcons	SE	MG	Sete Lagoas	Private	ComBr	0	1	0	0	–	Yes
27	Fundação Zoobotânica de Belo Horizonte	SE	MG	Belo Horizonte	Foundation	Zoo	2	1	0	0	–	Yes
28	Zoológico Vale Verde	SE	MG	Betim	Private	Zoo	0	0	1	0	–	No
29	Jardim Zoológico do Rio de Janeiro	SE	RJ	Rio de Janeiro	County/Concession	Zoo	1	1	0	0	–	Yes
30	Fazenda Itaoca	SE	SP	Indaiatuba	Private	ComBr	1	1	0	3	2007–2009	Yes
31	Fundação Parque Zoológico de São Paulo	SE	SP	São Paulo	Foundation	Zoo	2	2	0	0	–	Yes
32	Parque Ecológico de São Carlos	SE	SP	São Carlos	County	Zoo	1	1	0	0	–	Yes
33	Parque Ecológico Municipal Eng. Cid Almeida Franco	SE	SP	Americana	County	Zoo	1	1	0	0	–	Yes
34	Parque Zoológico Municipal Quinzinho de Barros	SE	SP	Sorocaba	County	Zoo	1	1	0	0	–	Yes
35	Zooparque de Itatiba	SE	SP	Itatiba	Private	Zoo	2	2	1	0	2012	Yes
36	Criadouro Onça Pintada	S	PR	Curitiba	Private	Zoo	1	0	0	1	–	Yes
37	Parque das Aves	S	PR	Foz do Iguaçu	Private	Zoo	1	1	0	0	–	Yes
38	Zoológico Municipal de Curitiba	S	PR	Curitiba	County	Zoo	0	1	1	1	–	Yes
39	Zoológico Roberto Ribas Lange – Itaipu Binacional	S	PR	Foz do Iguaçu	Private	Zoo	4	5	14	10	2006–2020	Yes
40	Hayabusa Falcoaria e Consultoria Ambiental	S	RS	São Francisco de Paula	Private	ComBr	1	2	0	0	–	Yes
						Total	35	51	29	24	–	–

The Convention on Biological Diversity (CDB 1993) and International Union for the Conservation of Nature (IUCN/SSC 2014) recognised that in situ conservation actions, such as captive breeding in zoos, will need to be combined with ex situ approaches. Some National Actions Plans for Species Conservation (hereafter PAN-Plano de Ação Nacional) that involve the Harpy Eagle have been implemented in many regions for which there are records of occurrence for this species in Brazil (Soares et al. 2008; Brasil 2012, 2014b, c, 2017). PAN for Birds of Prey Conservation (Soares et al. 2008) included, among the goals for the birds of prey captive population management, the identification of the number of individuals in Brazil and abroad, including information about the sex, individual identification (rings and microchips), time under human care, and origin. PAN of Amazonian Birds recommended integrated in situ and ex situ conservation programs (Brasil 2014b), as was stated in the IUCN guidelines about the use of ex situ management for species conservation (IUCN/SSC 2014). Seen in these terms, this article presents a revision of the Harpy Eagle ex situ population worldwide with a focus on Brazil.

## ﻿Materials and methods

‘Ex situ’ was used here as defined by the IUCN ex situ Guidelines, as conditions under which individuals are spatially restricted with respect to their natural spatial patterns or those of their progeny, are removed from many of their natural ecological processes, and are managed on some level by humans (IUCN/SSC 2014).

Some information about the ex situ population of Harpy Eagle was gathered from previous data available in environmental institutions, such as wildlife raptor centres, wildlife centres, the environmental police, animal institution keepers, and breeding centres, hereafter called ex situ facilities. Three methods were used to gather data.

### ﻿Available information and published data

A literature review took place to gather data about the ex situ facilities with records of Harpy Eagles under human care in Brazil and other countries. Moreover, three data sources were updated with information from researchers that work with this species in Brazil and had previously gathered data: Azeredo (2002, 2005), Banhos (2009), Banhos et al. (2016), and the Harpy Eagle Project database. The latter were collected from 2001 to 2020 during visits to Brazilian institutions keeping Harpy Eagles when personal communication, transcription from files, and interviews with employees took place. The Brazilian Institute of Environment and Renewable Natural Resources (**IBAMA**; Brazil 2019) database was also consulted using the Citizen Information Service Electronic System. Historic files were rare, although records from the early 1960’s were obtained. The results were compiled in two groups: before 1983 with the law legalising zoos in Brazil (Brasil 1983) and after 1984.

### ﻿Survey

A survey form was applied to 36 institutions keeping Harpy Eagles in Brazil (Suppl. material 1: Table S1). The queries included the number of individuals, gender, place of origin, and details in cases of rescued animals, including origin (from wild or bred in captivity under human care) and mating, with data up to 2020. The acronym names for all states that received the forms are as follows:

**AC** Acre;

**AM** Amazonas;

**AP** Amapá;

**BA** Bahia;

**CE** Ceará;

**DF** Distrito Federal;

**ES** Espírito Santo;

**GO** Goiás;

**MA** Maranhão;

**MG** Minas Gerais;

**PA** ParáPará;

**PE** Pernambuco;

**PI** Piauí;

**PR** Paraná;

**RJ** Rio de Janeiro;

**RS** Rio Grande do Sul;

**SE** Sergipe;

**SP** São Paulo.

There were another five institutions keeping Harpy Eagles that were contacted after the surveys were finished. They did not receive the survey form, but their information was included in the results.

### ﻿Zoological Information Management Software database

The Species360 Zoological Information Management Software (**ZIMS**) database (ZIMS 2020) was consulted. Additionally, experts in birds of prey were asked to identify institutions that had ex situ populations of Harpy Eagles outside Brazil, and the Harpy Eagle Project collection database was referred to for visits to institutions outside Brazil, in Panama, Ecuador, Argentina, and French Guiana (2014–2018).

## ﻿Results

### ﻿Ex situ Harpy Eagle population in Brazil

#### Survey form

Twenty-nine (72.5%) institutions keeping Harpy Eagles ex situ answered the survey form, which provided institutional results to be combined with the information from other sources. Seven (17.5%) institutions do not return the survey answered (Table 1), with exception of the Crax Sociedade de Pesquisa de Fauna Silvestre, all other facilities have one individual or a pair.

### ﻿Entrance of Harpy Eagles to ex situ facilities

#### Harpy Eagles from the wild

**Figure 1.**
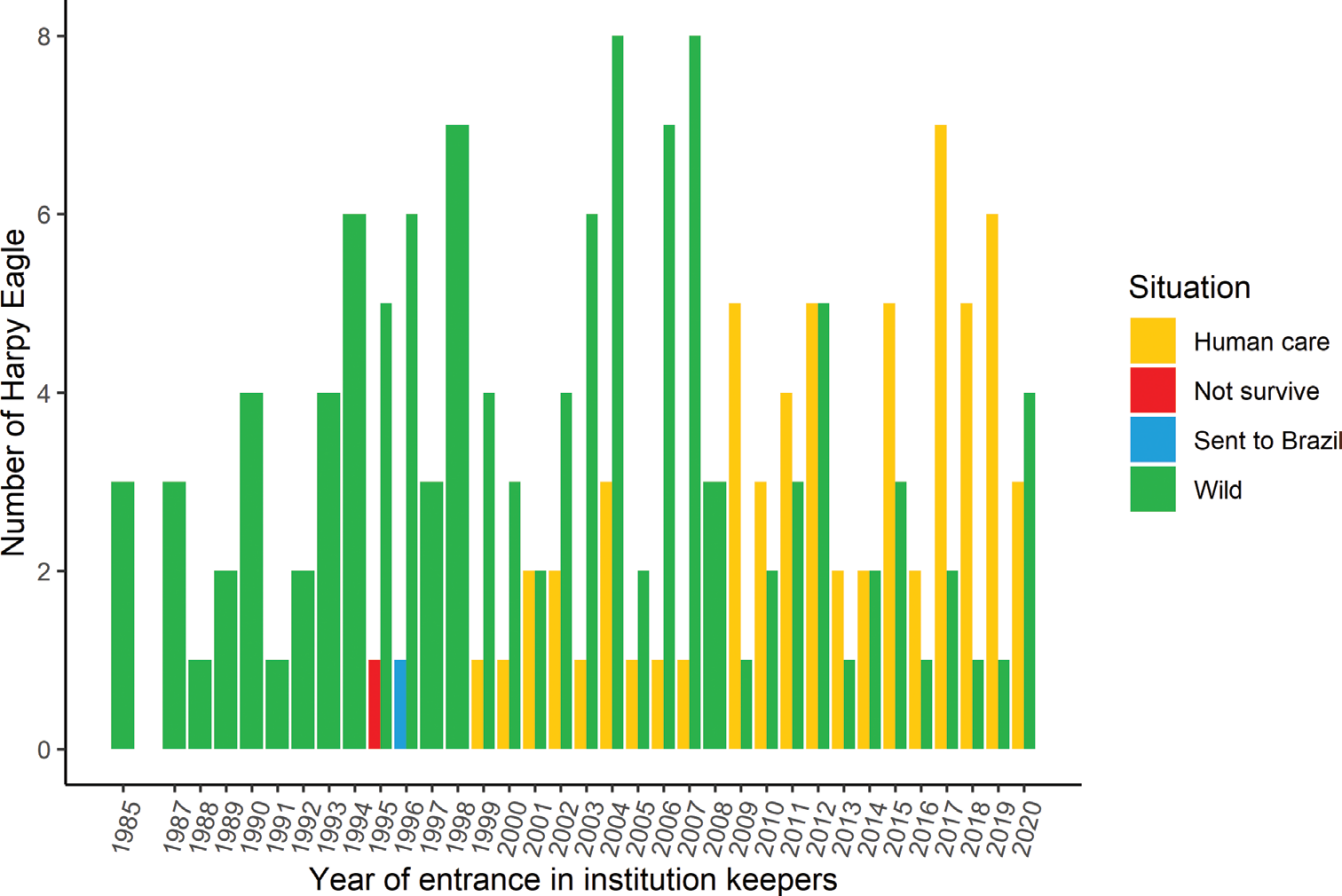
Number of Harpy Eagles (*Harpiaharpyja*) yearly entrance to Brazilian institutions between 1984 and 2020.

Thirteen records came from documented reports from 1963 to 1983 (20-years period), referring to one individual in 1966, 1972, and 1980 and two individuals in 1963, 1973, 1975, and four individuals 1979. Of those 13 individuals, only one was still alive in 2020. In the last 37 years (1984 to 2020), it was possible to document a minimum of 122 wild Harpy Eagle entrances to facilities in Brazil, with an average of 3.2 individuals/year. The highest entrance rate was nine individuals in 2004 and 2007 (Fig. 1). Of those 122 wild individuals, 35 died before the initiation of this study.

#### Harpy Eagles bred in captivity under human care

The first record of Harpy Eagle breeding under human care in Brazil was in 1995 by the former conservation breeder Erico Albuquerque de Abreu e Lima; however, the chick did not survive (Azeredo 2005; Fig. 1). In 1988, one Harpy Eagle hatched and was bred in captivity under human care in Germany. In 1996, it was sent to Brazil to the care of the Society of Research and Wildlife – CRAX (R Azeredo pers. comm.; Globo Rural 2012; Fig. 1). The first successful captive breeding in Brazil occurred in 1999 by CRAX (Azeredo 2005). Sixty-two individuals hatched under human care in the period 1999–2020 (Fig. 1). Of those 62 individuals, six died and four were sent to institutions in Europe.

#### Harpy Eagle ex situ population in 2020

In 2020, the Harpy Eagle ex situ population in Brazil comprised 139 individuals kept in 40 institutions (Table 1), of which 86 (62%) were taken from the wild (35 males and 51 females), while 53 (38%) hatched and were bred in captivity under human care (29 males and 24 females; Table 1). On average (±SD), the institutions kept 2.2 ± 1.4 Harpy Eagles, not including two institutions, CRAX Society of Research and Wildlife and Roberto Ribas Lange Zoo, that kept 21 and 33 individuals, respectively (Table 1).

#### Wild Harpy Eagle locality of origin

**Figure 2.**
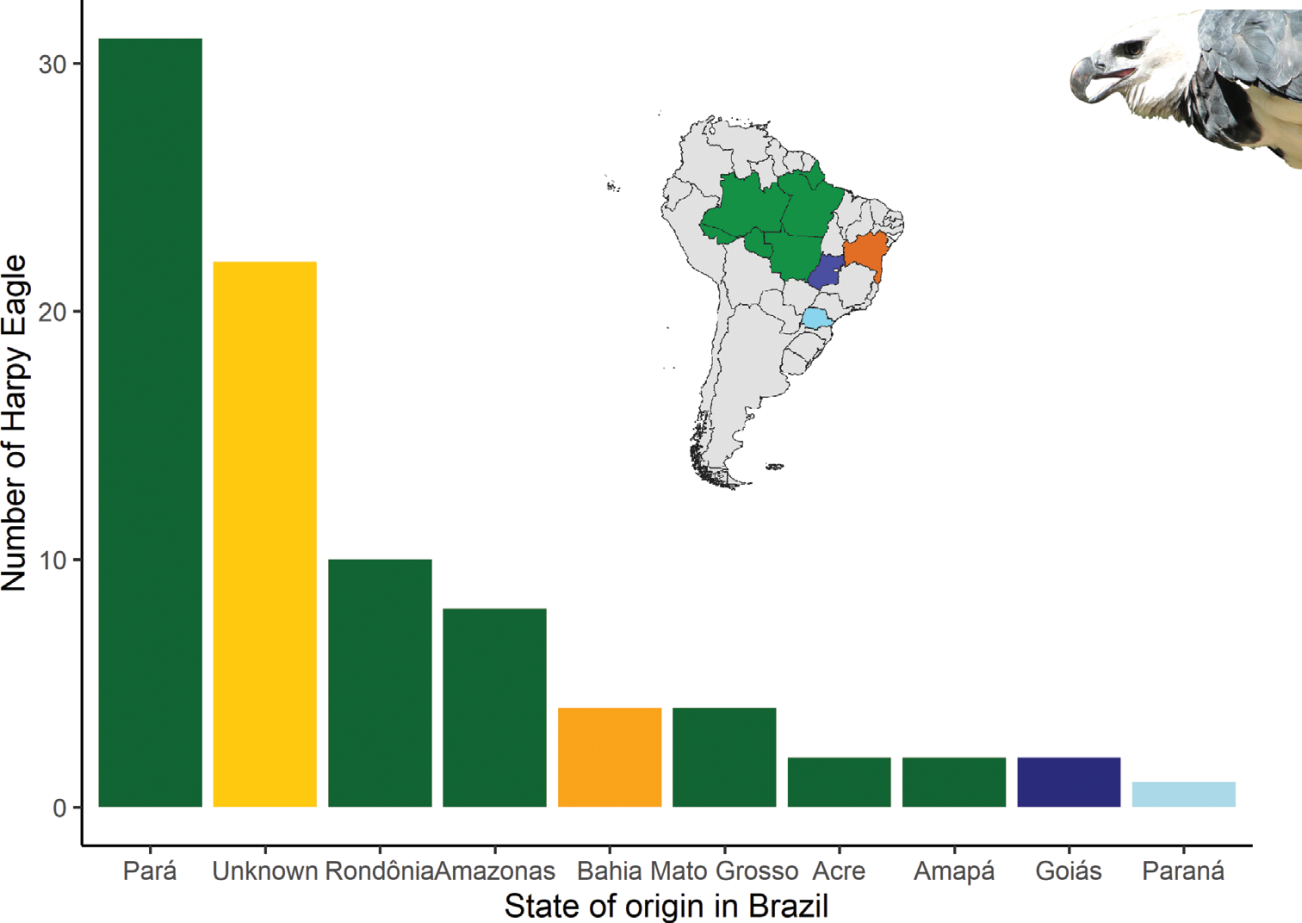
Origin of wild Harpy Eagles (*Harpiaharpyja*) by state and biome kept in Brazilian ex situ facilities in 2020.

Of the 86 wild Harpy Eagles, 64 (74%) individuals had a known state of origin, and 22 (26%) individuals were of unknown origin. Most Harpy Eagles came from the Amazon biome, Pará state (*n* = 31; 35%), followed by Rondônia state (*n* = 10; 11%) and Amazonas state (*n* = 8; 9%), Mato Grosso state (*n* = 4; 5%), Acre state (*n* = 2; 2%), and Amapá state (*n* = 2; 2%). In addition to the Amazon biome, other biomes were also the source of wild Harpy Eagles, including the Brazilian Atlantic Forest, Bahia state (*n* = 4; 4%) and Paraná state (*n* = 1; 1%). The Cerrado had two (2%) individuals from Goiás state (Fig. 2).

#### Type of entrance from nature to the first facility

**Figure 3.**
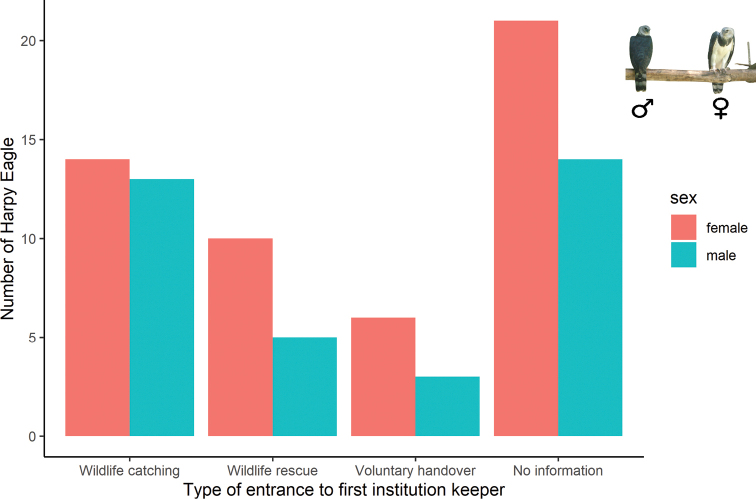
Type of entrance of wild Harpy Eagles (*Harpiaharpyja*) to their first ex situ facility in Brazil.

The source of their entrance to the first facility was possible to determine for 53 (60%) individuals only due to the lack of information. Records were classified as wildlife catching (33%), wildlife rescue (17%), and voluntary handover (10%). Females were the majority in all categories (Fig. 3).

#### Location, management category, and administration type of the institution

Geographic locations for the 40 Harpy Eagle facilities in Brazil were mainly in the Southeast (*n* = 14) and North regions (*n* = 10), with seven in the northeast, five in the south, and four in the midwest (Table 1). Of the facilities, 60% were under private administration, followed by state (40%), county (15%), federal (7.5%), and foundation administration (7.5%; Table 1). The majority of these ex situ facilities were registered in the management category as zoos (*n* = 24; 60%), followed by conservation breeders (*n* = 7; 17.5%), commercial breeders (*n* = 6; 15%), wildlife shelters (*n* = 2; 5%), and wildlife sorting centre (*n* = 1; 2.5%; Table 1).

Among those institutions, 23 kept one Harpy Eagle pair or more individuals, while other 17 institutions kept only single. Within the institutions with one gender, there were ten zoos (11 females and 2 males), one commercial breeder (1 female), three conservation breeding (3 females and 3 males), two wildlife shelters (2 females), and one wildlife sorting centre (IBAMA; 2 females; Table 1).

#### Harpy Eagle breeding records under human care

Based only on the survey information, seven institutions had some attempt or success of captive breeding from 1999 to 2020 (Table 1). Three other ex situ facilities that no longer exist had breeding success: the conservation breeder Tropicus in the Rio de Janeiro municipality (2001), the conservation breeder Erico Albuquerque de Abreu e Lima in the Distrito Federal municipality (2005), and the breeder Parque da Varginha in the Tocantins municipality (2010). The three individuals kept in these institutions were transferred to active ex situ facilities.

### ﻿Ex situ Harpy Eagle population outside Brazil

**Figure 4.**
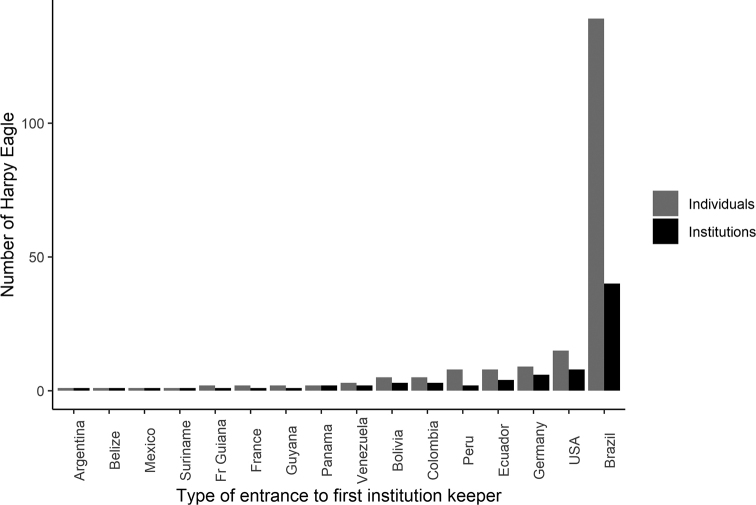
Harpy Eagles (*Harpiaharpyja*) ex situ population worldwide in 2020.

In 2020, there were 66 Harpy Eagles kept in 37 facilities outside Brazil, distributed among 15 countries, representing 32% of the entire ex-situ population (68% were in Brazil) and 48% of all ex situ facilities (52% were in Brazil; Fig. 4). The United States, Germany, Peru, Ecuador, and Colombia had the largest number of individuals (Fig. 4). Of those 66 Harpy Eagles, 36 were males and 29 were females, and in one, the sex was unknown. In South America, 26 came from the wild (13 males and 13 females), one male was bred in captivity under human care, and eight were of unknown origin. In Central America, two females came from the wild and one male was bred in captivity under human care. In North America, two males came from the wild, 14 were bred in captivity under human care (7 males and 7 females). In Europe, 11 were bred in captivity under human care (7 males and 4 females), and one was of unknown origin (Suppl. material 2: Table S2).

## ﻿Discussion

Lack of data regarding the capture method and place of origin (locality) of the Harpy Eagles imposed a challenge to information collection in this study. In most cases, there was a lack of data on the records of wildlife catching and rescue centres, contributing to a high number of unknown origin localities. Additionally, much information was lost when Harpy Eagles were transferred between institutions.

The first Harpy Eagle reported in the care of a zoo in Brazil was in 1895, at the Parque Zoobotânico do Museo Goeldi, in the state of Pará, one of the oldest zoos in the country (Sanjad et al. 2012). This same zoo received a second individual in 1901 (Sanjad et al. 2012). The voucher specimen MPEG3445 conserved by taxidermy from the “Fernando Novaes” bird collection at Museo Paraense Emílio Goeldi, dated from 1904, probably belonged to one of those birds. A small private zoo, Jardim Zoológico Vila Isabel in Rio de Janeiro, also held a Harpy Eagle, which died sometime between 1890 and 1899 and was donated to the Museo Nacional (Pais 2013). Of the three oldest recorded individuals, two of them entered the Zoológico de Goiânia in 1963; both came from Roraima state (José Hidasi, pers. comm). One of these birds was taxidermically conserved (skin voucher N.13.268) in the bird collection at Fundação Museo de Ornitologia. The third Harpy Eagle entered the Zoológico de Brasília in 1966 but died in 1969 with no further information.

The compilation from 1963 to 1983 did not reflect a precise quantity due to the lack of recorded files at the majority of institutions, which were our information sources. However, the compilation from 1984 to 2020 was well recorded and revealed a high rate of individuals removed from nature (3.2 individuals/year), including bird victims from conflict between birds and humans in Brazil. In 2020, four Harpy Eagles entered Brazilian ex situ facilities; this number is four times greater than the previous two years, and it was the highest number since 2012 (Fig. 1). Notably, in 2020, the global COVID-19 pandemic began and had strong impacts on people’s health and activities; however, it also impacted biodiversity due to the reduction in protection and inspection of natural habitats (Bang and Khadakkar 2020; Corlett et al. 2020), and apparently the Harpy Eagle was not exempt from this effect. Although the type of conflicts involved has not been documented for the majority of cases, in general, individuals removed from nature and sent to these ex situ facilities were attained after suffering from collision traumas with vehicles, falling from tree-nests, being injured by gunshot, receiving wounds of uncertain origin and being kept in captivity illegally by people (Soares et al. 2008; Amorim et al. 2010; Silva et al. 2010). The retention of Harpy Eagle individuals that were alive but could not return to the wild, in addition to the number that were killed by hunters (Trinca et al. 2008; Giraldo-Amaya et al. 2021) and other sources of human-animal conflict (Gusmão et al. 2020), most likely contributed to the populational decline of the Harpy Eagle in the wild. Harpy Eagles transferred from the wild into zoos were mainly females (59%), which may have impacted the demography of the wild Harpy Eagle population.

The Amazon has been the major source of Harpy Eagles that have been removed from nature, and the states of origin within the region are mainly Para, Rondônia, Amazonas, and Mato Grosso, which have experienced the world’s highest absolute rate of forest destruction (Soares-Filho et al. 2006; Silva Junior et al. 2021). Likewise, the Brazilian Atlantic Forest was reduced and fragmented (Ribeiro et al. 2009), and there have been few records about free-ranging Harpy Eagles (Aguiar-Silva et al. 2012; Meller and Guadagnin 2016). The Brazilian Atlantic forests have still been subject to Harpy Eagle removal from nature, with five individuals taken from Bahia state in the northeast region (Table 1). One individual was removed from the Biome Cerrado. In this biome, Harpy Eagle records have been always rare and this corresponds to this comparably low number. However, there were more records available in gallery forests and in the transition into the Amazonia (Pinheiro and Dornas 2009; Silva et al. 2013; Pascoal et al. 2014) and the Atlantic Forest (Oliveira and Silva 2006; Pereira and Salzo 2006).

For Harpy Eagle conservation, the return of all captured Harpy Eagles in adequate health conditions back to nature, re-establishing these individuals into the natural population, is a complex process but one that is necessary for a health and functional ecosystem. There exists a decision tree to assist the assessment of birds in Brazil at stages of the process after a rescue of injured individuals or from trades and from illegal traffic or captivity (Efe et al. 2006). In 2018, the Harpy Eagle Project in Brazil (Projeto Harpia) began a national network to rehabilitate those Harpy Eagles; however, rehabilitation through the network has not yet been possible; in most cases, this was declared to be due to lack of funds, staff, and specific infrastructure, although the Project has succeeded with a number of individuals in the past for specific regions in the country. The idea of this network is to use the zoo’s support during the rehabilitation of the individuals. If rehabilitation is not possible, individuals may be allocated to an ex situ conservation program. An integrated in situ and ex situ conservation program, involving the rehabilitation of animals removed from nature seems necessary. There is an increasing need for a ‘one plan approach’ to develop multi-disciplinary conservation strategies that include the integration of in situ and ex situ management processes (Byers et al. 2013).

Captive breeding can play a crucial role in the recovery of some species for which effective alternatives are unavailable in the short term, while protecting species habitats and ecosystems (Collar and Butchart 2014; Fleming et al. 2011; Snyder et al. 1996). Breeding under human care has the potential to maintain targeted populations as an ‘insurance policy’ against threats until reintroduction into the wild is possible (Conde et al. 2011). Breeding aimed at the restoration of populations in danger of extinction must not be replaced with breeding for other goals such as exposition, conservationist education, or research (Snyder et al. 1996). However, zoos have played an important role in the conservation of endangered species by promoting and supporting environmental awareness, providing professional qualifications, and facilitating research and in situ conservation programs to support environmental recovery projects (Snyder et al. 1996; Conde et al. 2011). Moreover, zoos contributed to species conservation and increased interest and public affection by reporting the success of captive breeding and by educating the public about the importance of a high biodiversity (Gusset and Dick 2010; Zimmermann 2010). In Brazil, for example, the Roberto Ribas Lange Zoo, in a five-year period (2012 to 2017), was visited by 146,633 visitors (Cubas et al. 2017). Zoos worldwide are providing conservation funds, investing millions in ex situ and in situ wildlife activities (Gusset and Dicke 2010; Zimmermann 2010; Fa et al. 2014). Assisted reproduction may be of high future for ex situ conservation and should be considered in future as part of a multidisciplinary one-plan approach for the conservation of Harpy Eagles (Blanco et al. 2002a, b; Gee et al. 2004; Fischer et al. 2014).

Harpy Eagle ex situ populations outside Brazil consisted of 35 wild individuals, most in South America. An important step is to understand their characteristics as a source of genetic diversity. Currently, some of those zoos in Europe that have Harpy Eagle individuals hatched in captivity are contributing to ex situ and in situ conservation of Harpy Eagles by promoting funds for research, for example, ZooParc de Beauval (France) and Tiergarten Nürnberg (Germany).

To reach the ex situ conservation goals as required by article 9 of the CBD (Glowka et al. 1994; Conde et al. 2011), future conservation activities should be focused on joining forces and acting in an integrated manner for handling. The establishment of a structured ex situ Harpy Eagle program and an international Harpy Eagle studbook seems to be required. The potential roles of a species conservation program must be clearly defined and should include the maintenance of a healthy and genetically diverse ex situ backup population, measures to rescue and rehabilitate wild individuals, population restoration, research, training, and education in accordance with the IUCN guidelines (McGowan et al. 2017).

## ﻿Conclusions

Brazil maintains the largest ex situ Harpy Eagle population in the world. Brazilian institutions played an important role in breeding for ex situ conservation of the Harpy Eagle. A great number of institutions in South and Central America keep wild individuals, while North America and Europe mainly keep individuals bred in captivity under human care. Information about ex situ individuals must be incorporated into a studbook for Harpy Eagle population management. These individuals may potentially constitute a genetically and demographically viable backup population for future conservation attempts, as well as a source of research and education applied to Harpy Eagle conservation. The Harpy Eagle ex situ population must be used in integrated planning to support in situ population conservation.

## Supplementary materials

Supplementary material 1 Table S1 Data type docx. file Explanation note Survey form sent to 40 Harpy Eagles (Harpiaharpyja) ex situ facilities in Brazil. https://binary.pensoft.net/file/637686This dataset is made available under the Open Database License (http://opendatacommons.org/licenses/odbl/1.0/). The Open Database License (ODbL) is a license agreement intended to allow users to freely share, modify, and use this Dataset while maintaining this same freedom for others, provided that the original source and author(s) are credited.Marcos José de Oliveira, Francisca Helena Aguiar-Silva, Wanderlei de Moraes, Tânia Margarete Sanaiotti, Aureo Banhos, Nei Moreira

Supplementary material 2 Table S2 Data type docx. file Explanation note Harpy Eagle (Harpiaharpyja) ex situ population outside Brazil in 2020. SAm-South America, CAm-Central America, NAm-North America, EU-Europe. https://binary.pensoft.net/file/637687This dataset is made available under the Open Database License (http://opendatacommons.org/licenses/odbl/1.0/). The Open Database License (ODbL) is a license agreement intended to allow users to freely share, modify, and use this Dataset while maintaining this same freedom for others, provided that the original source and author(s) are credited.Marcos José de Oliveira, Francisca Helena Aguiar-Silva, Wanderlei de Moraes, Tânia Margarete Sanaiotti, Aureo Banhos, Nei Moreira
