# Supplementary material for: ﻿Ex situ population of the Harpy Eagle and its potential for integrated conservation
Source: Zookeys. 2022 Jan 25;1083:109–28. doi: 10.3897/zookeys.1083.69047 (PMC8807570; doi:10.3897/zookeys.1083.69047)
Supplement: Supplementary material 1 — Table S1 [file zookeys-1083-109-s001.docx]

# Supplementary Table 1. Survey form sent to 36 Harpy Eagles (*Harpia harpyja*) ex situ facilities in Brazil.

| Institution: | | | | | | | | | | | | | | |
| --- | --- | --- | --- | --- | --- | --- | --- | --- | --- | --- | --- | --- | --- | --- |
| Person in charge: | | | | | | | | | | | | | | |
| E-mail: | | | | | | | | | | | | | | |
| Phone number: | | | | | | | | | | | | | | |
| Administration type: | | | | | | | | | | | | | | |
| **Harpy Eagle (HA) Collection Data** | | | | | | | | | | | | | | |
| HA ID number | Sex | Age* | | Arrival date | Hatching date | | | Mother’s ID (if known) | | Father’s ID (if known) | Wild or bred in captivity | Institution of origin** | | Capture method*** |
| *Inform the age and the color of the Harpy Eagle’s feathers in the moment of its arrival in the institution.  **The institution of origin may include wildlife centers, wildlife shelters, wildlife screening centers, Animal Rescue and Care Companies, Zoos, Commercial and Conservationist Breeding Centers.  ***The origin of the Harpy Eagle was via donation, seized from traffic, exchange, loan or animal legal custody. | | | | | | | | | | | | | | |
| **Information about the Harpy Eagle pairs**  Fill this formulaire for each Harpy Eagle pair. | | | | | | | | | | | | | | |
| Pair identification: | | | | | | | | | Enclosure number | | | | Pairing date | |
| Male Identification: | | | | | | | | |  |  |  |  |  |  |
| Female Identification: | | | | | | | | |  |  |  |  |  |  |
| Laying date | | | Fertile eggs (y/n) | | | Embryonic death | | | Disappeared or predated eggs | | | | Hatching date | |
| **Permission for the use of this information** | | | | | | | | | | | | |  | |
| The Institution allows the use of this information in the Marcos José de Oliveira Master’s dissertation – Programa de Pós-Graduação em Zoologia in the Federal University of Parana - UFPR, citing the authors. | | | | | | | | | | | | | ( ) Yes  ( ) No | |
|  |  |  |  |  |  |  |  |  |  |  |  |  |  |  |
|  |  |  |  |  |  |  |  |  |  |  |  |  |  |  |
| Date: | | | | | | | Signature: | | | | | | | |
|  |  |  |  |  |  |  |  |  |  |  |  |  |  |  |
