# Supplementary material for: ﻿Ex situ population of the Harpy Eagle and its potential for integrated conservation
Source: Zookeys. 2022 Jan 25;1083:109–28. doi: 10.3897/zookeys.1083.69047 (PMC8807570; doi:10.3897/zookeys.1083.69047)
Supplement: Supplementary material 2 — Table S2 [file zookeys-1083-109-s002.docx]

**Supplementary Table 2** – Harpy Eagle (*Harpia harpyja*) *ex situ* population outside Brazil in 2020. SAm—South America, CAm—Central America, NAm—North America, EU—Europe. M – Male, F – Female.

| # | Institution Keeper | Region | Country | Locality | Origin of Birth | | | | Unknown Sex and/ or Origin |
| --- | --- | --- | --- | --- | --- | --- | --- | --- | --- |
|  |  |  |  |  | Wild | | Bred in Captivity | |  |
|  |  |  |  |  | ♂ | ♀ | ♂ | ♀ |  |
| 1 | Zoo Bal Park de Montecarlo | SAm | Argentina | Montecarlo | 1 | 0 | 0 | 0 |  |
| 2 | Zoológico Municipal Noel Kempff Mercado | SAm | Bolivia | Sta Cruz de La Sierra | 0 | 2 | 0 | 0 |  |
| 3 | Centro de Atención y Derivación de Fauna Silvestre (CAD) | SAm | Bolivia | Sta. Cruz de La Sierra | 1 | 1 | 0 | 0 |  |
| 4 | Zoológico Vesty Pakos | SAm | Bolivia | La Paz | 1 | 0 | 0 | 0 |  |
| 5 | Zoológico de Barranquilha | SAm | Colombia | Barranquilla | 0 | 1 | 0 | 0 |  |
| 6 | Aviário Nacional | SAm | Colombia | Cartagena | - | 0 | - | 0 | One male |
| 7 | Bioparque La Reserva | SAm | Colombia | Cota | 1 | 2 | 0 | 0 |  |
| 8 | Parque Condor | SAm | Ecuador | Otavalo | 0 | 1 | 1 | 0 |  |
| 9 | Parque Histórico de Guayaquil | SAm | Ecuador | Guayaquil | - | - | - | - | Two males; one female |
| 10 | Zoológico El Pantanal | SAm | Ecuador | Guayaquil | - | 0 | - | 0 | One male |
| 11 | Hillary Nature Resort & Spa | SAm | Ecuador | Arenillas | - | - | - | - | One male; one female |
| 12 | Zoo de Guyana (Macouria) | SAm | French Guiana | Macouria | 2 | 0 | 0 | 0 |  |
| 13 | Guyana Zoological Park | SAm | Guyana | Georgetown | 1 | 1 | 0 | 0 |  |
| 14 | Coalisión por la Amazonia | SAm | Peru | Tambopata | 1 | 0 | 0 | 0 |  |
| 15 | Zoocriadero el Huayco | SAm | Peru | Lima | 3 | 4 | 0 | 0 |  |
| 16 | Zoológico de Paya Leslie Pantin | SAm | Venezuela | Santiago Mariño | 1 | 0 | 0 | 0 |  |
| 17 | Zoológico Las Delícias | SAm | Venezuela | Maracay | 1 | 1 | 0 | 0 |  |
| 18 | Zoological Garden Paramaribo (Zoologie Parc of Paramaribo) | SAm | Suriname | Paramaribo | - | - | 0 | 0 | One bird from wild |
| 19 | The Belize Zoo | CAm | Belize | Belmopan | 0 | 0 | 1 | 0 |  |
| 20 | Parque Municipal Summit | CAm | Panama | Cidade do Panamá | 0 | 1 | 0 | 0 |  |
| 21 | Zoológico del Istmo | CAm | Panama | Cidade do Panamá | 0 | 1 | 0 | 0 |  |
| 22 | Dallas Zoo | NAm | USA | Dallas | 0 | 0 | 0 | 2 |  |
| 23 | Dallas Wordl Aquarium | NAm | USA | Dallas | 0 | 0 | 0 | 1 |  |
| 24 | Los Angeles Zoo & Botanical Garden | NAm | USA | Los Angeles | 0 | 0 | 1 | 1 |  |
| 25 | Miami Zoo | NAm | USA | Miami | 0 | 0 | 2 | 1 |  |
| 26 | San Diego Zoo | NAm | USA | San Diego | 0 | 0 | 1 | 0 |  |
| 27 | Natural Encounters, Inc. Worldbird | NAm | USA | Winter Haven | 0 | 0 | 1 | 1 |  |
| 28 | Fort Worth Zoo | NAm | USA | Fort Worth | 1 | 0 | 1 | 1 |  |
| 29 | Crawford W | NAm | USA | Crawford | 0 | 0 | 1 | 0 |  |
| 30 | El Nido Aviário | NAm | Mexico | Ixtapaluca | 1 | 0 | 0 | 0 |  |
| 31 | Tierpark Berlin | EU | Germany | Berlin | 0 | 0 | 1 | 0 |  |
| 32 | Nuremberg Zoo (Tiergaten) | EU | Germany | Nurnberg | 0 | 0 | 3 | 2 |  |
| 33 | Weltvogelpark | EU | Germany | Walsrode | - | 0 | - | 0 | One male |
| 34 | Zoologischer Garten Wuppertal | EU | Germany | Wuppertal | 0 | 0 | 0 | 1 |  |
| 35 | Schilling | EU | Germany | Schilling | 0 | 0 | 1 | 0 |  |
| 36 | Dortmund_Zoo | EU | Germany | Dortmund | 0 | 0 | 1 | 0 |  |
| 37 | Zoo Parc de Beauval | EU | France | Beauval | 0 | 0 | 1 | 1 |  |
|  |  |  |  | TOTAL | 15 | 15 | 16 | 11 | 9 |
